# Mobile Health Apps That Help With COVID-19 Management: Scoping Review

**DOI:** 10.2196/20596

**Published:** 2020-08-06

**Authors:** Hanson John Leon Singh, Danielle Couch, Kevin Yap

**Affiliations:** 1 Department of Public Health School of Psychology and Public Health La Trobe University Melbourne Australia; 2 School of Rural Health Faculty of Medicine, Nursing and Health Sciences Monash University Bendigo Australia

**Keywords:** COVID-19, mobile apps, mHealth, contact tracing, symptom monitoring, information provision, mobile health

## Abstract

**Background:**

Mobile health (mHealth) apps have played an important role in mitigating the coronavirus disease (COVID-19) response. However, there is no resource that provides a holistic picture of the available mHealth apps that have been developed to combat this pandemic.

**Objective:**

Our aim is to scope the evidence base on apps that were developed in response to COVID-19.

**Methods:**

Following the PRISMA (Preferred Reporting Items for Systematic Reviews and Meta-Analyses) guidelines for scoping reviews, literature searches were conducted on Google Search, Google Scholar, and PubMed using the country’s name as keywords and “coronavirus,” “COVID-19,” “nCOV19,” “contact tracing,” “information providing apps,” “symptom tracking,” “mobile apps,” “mobile applications,” “smartphone,” “mobile phone,” and “mHealth.” Countries most affected by COVID-19 and those that first rolled out COVID-19–related apps were included.

**Results:**

A total of 46 articles were reviewed from 19 countries, resulting in a total of 29 apps. Among them, 15 (52%) apps were on contact tracing, 7 (24%) apps on quarantine, 7 (24%) on symptom monitoring, and 1 (3%) on information provision. More than half (n=20, 69%) were from governmental sources, only 3 (10%) were from private organizations, and 3 (10%) from universities. There were 6 (21%) apps available on either Android or iOS, and 10 (34%) were available on both platforms. Bluetooth was used in 10 (34%) apps for collecting data, 12 (41%) apps used GPS, and 12 (41%) used other forms of data collection.

**Conclusions:**

This review identifies that the majority of COVID-19 apps were for contact tracing and symptom monitoring. However, these apps are effective only if taken up by the community. The sharing of good practices across different countries can enable governments to learn from each other and develop effective strategies to combat and manage this pandemic.

## Introduction

The novel coronavirus, severe acute respiratory syndrome coronavirus 2 (SARS-CoV-2) [1], manifests the coronavirus disease (COVID-19) and was first identified in Wuhan, China in December 2019 [2]. It first presented as severe cases of pneumonia of unknown origin and was identified as a coronavirus in January 2020 [3]. SARS-CoV-2 affects individuals of all ages and spreads through droplets when the infected individuals cough or sneeze [3]. The droplets can still be infectious even after deposition onto surfaces. Infection occurs when these droplets are inhaled or when the contaminated surfaces are touched followed by the touching of one’s eyes, nose, or mouth. Transmission of infection is possible during its incubation phase (2-14 days), and common symptoms include fever, cough, sore throat, headache, myalgia, fatigue, and breathlessness [3]. The symptoms and manifestations vary greatly among individuals; some have serious consequences like acute respiratory distress syndrome and organ failure, while others can be asymptomatic [3]. Older adults and individuals with comorbidities like diabetes, hypertension, and cardiovascular problems are more susceptible and can manifest more severe symptoms when infected [4].

Since the public announcement of the first few cases, SARS-CoV-2 has spread worldwide and was declared as a pandemic on March 11, 2020, by the World Health Organization (WHO) [5]. As of August 1, 2020, there were over 17.9 million cases recorded and over 680,000 deaths due to COVID-19 [6]. As COVID-19 spread worldwide into countries with different health systems and responses, the number of infected cases constantly changed. To control its spread, several prevention strategies were adopted by various countries. These strategies included self-isolation and quarantine for individuals who were suspected cases of infection or showed mild symptoms [3], wearing of face masks, and adherence to hygienic practices [3]. Public gatherings were also avoided to limit the number of close contacts among individuals [3]. During the first wave of COVID-19, various mobile health (mHealth) apps were rapidly developed in response to tackle the virus.

The first COVID-19 apps that were developed and widely publicized were contact tracing apps, which were created to notify its users if they had crossed paths with another person infected with the coronavirus [7]. The first national app was developed in Singapore, which used Bluetooth technology for contact tracing [8]. If someone was in close proximity with an infected individual, the app would send a push notification to alert them of possible COVID-19 infection and further suggest that they undergo testing [9]. The technology was made open source and shared internationally for other countries to build similar apps for their own populations [10-12]. Since then, there have been various other types of contact tracing apps available, each using different methods of data collection to track the movements of its users.

Symptom monitoring apps have also emerged in response to COVID-19. These apps commonly collect information about the user’s health by posing a list of questions related to symptom identification, from which a differential diagnosis is made [13]. However, other innovative methods have also been used, such as automatic collection and recording of the user’s health-related data (eg, temperature and pulse rates) from wearables like wristbands [14,15]. In the case of a suspected COVID-19 infection, the user is alerted and advised to go for a checkup at a nearby clinic.

The importance of credible information that can be provided in a timely manner to the public has in part been addressed by some of the information providing apps developed for COVID-19. Information providing apps give details about the coronavirus, disease, good hygiene practices, and guidelines to follow, like social distancing and the importance of wearing face masks [16-19]. However, during the initial stages of the pandemic, the mHealth markets saw the emergence of developers who were trying to take advantage of the situation by creating fake apps [20], as well as ransomware apps that mandated users to transfer money and threatened deletion of the phone’s storage if money was not transferred [21]. There were also large amounts of misinformation on the internet [22]. In response, the WHO worked with Google as well as popular social media sites like Facebook, Twitter, Tencent, and TikTok to combat this misinformation [23]. Furthermore, steps were taken by social media apps like Facebook, YouTube, Twitter, Instagram, and Snapchat to limit the rapid spread of misinformation to their massive audience reach [24]. In addition, Apple and Google made efforts to regulate COVID-19–related apps released in their app stores, only allowing apps developed by credible organizations [25]. WhatsApp, a popular communication app, also limited the number of times users could forward messages related to COVID-19 to reduce the spread of misinformation about the coronavirus [26].

Amid the rapidly evolving COVID-19 environment, mHealth apps have been playing an important role in mitigating the COVID-19 response, but to date, there has not been any overview and comparisons of the mHealth apps that have been developed to combat this pandemic. The aim of this review is to scope the evidence base for articles that described apps that were developed in response to the COVID-19 pandemic. This paper categorizes and compares the available apps by providing a description of these apps, their purposes, and the features employed. A recommendation of useful features is also provided for developers and interested stakeholders.

## Methods

This review was conducted following the guidelines of the PRISMA (Preferred Reporting Items for Systematic Reviews and Meta-Analyses) extension for scoping reviews [27]. The search period for information was from March 25, 2020, to May 5, 2020, when the outbreak rapidly spread to all parts of the world, including the United States [28]. Google search, Google Scholar, Scopus, and PubMed were used to find apps specific for each country. Considering the rapid pace of articles being published on COVID-19, Google Scholar, Scopus, and PubMed were selected, as these were common databases used by clinicians and would encompass a broad scope of journal articles that would be relevant to clinical and public health practices. The keywords used were: “coronavirus,” “COVID-19,” “nCOV19,” “contact tracing,” “information providing apps,” “symptom tracking,” “mobile apps,” “mobile applications,” “smartphone,” “mobile phone,” and “mHealth,” and the country names from the top 10 countries that had the greatest number of COVID-19 cases as of April 27, 2020. These countries were China, France, Germany, Iran, Italy, Russia, Spain, Turkey, the United Kingdom, and the United States [6]. Another 9 countries were added to the list, as they were among the first in releasing apps specific for COVID-19 based on articles found during the initial stages of the research from the period of March 25, 2020, to April 1, 2020. These countries included Hong Kong, Iceland, India, Indonesia, Malaysia, Poland, Singapore, South Korea, and Taiwan.

Due to the rapidly changing nature of the COVID-19 pandemic, the main sources of evidence were mainly from online news articles. Information from government websites and health departments of different countries were also reviewed as they were considered to be credible sources and would have the most up-to-date information available for the country [1,5,13,29,30]. Selected articles were limited to the first 10 pages of the Google search results, after which the articles were found to be irrelevant to COVID-19. All articles included in our review were after December 9, 2019, which was the day when the first infection was reported in China [2].

The inclusion criteria were articles that had a clear description about the features used in the apps. Articles from December 9, 2019, to May 5, 2020, were included. Apps that were not relevant to the disease and those that were in a language other than English were excluded. If the articles had limited information about the app, such as type of feature used, a second Google search was conducted with the specific app-related parameters to source for further information. This ensured that a complete profile of each app was obtained.

## Results

### Overview

**Figure 1:**
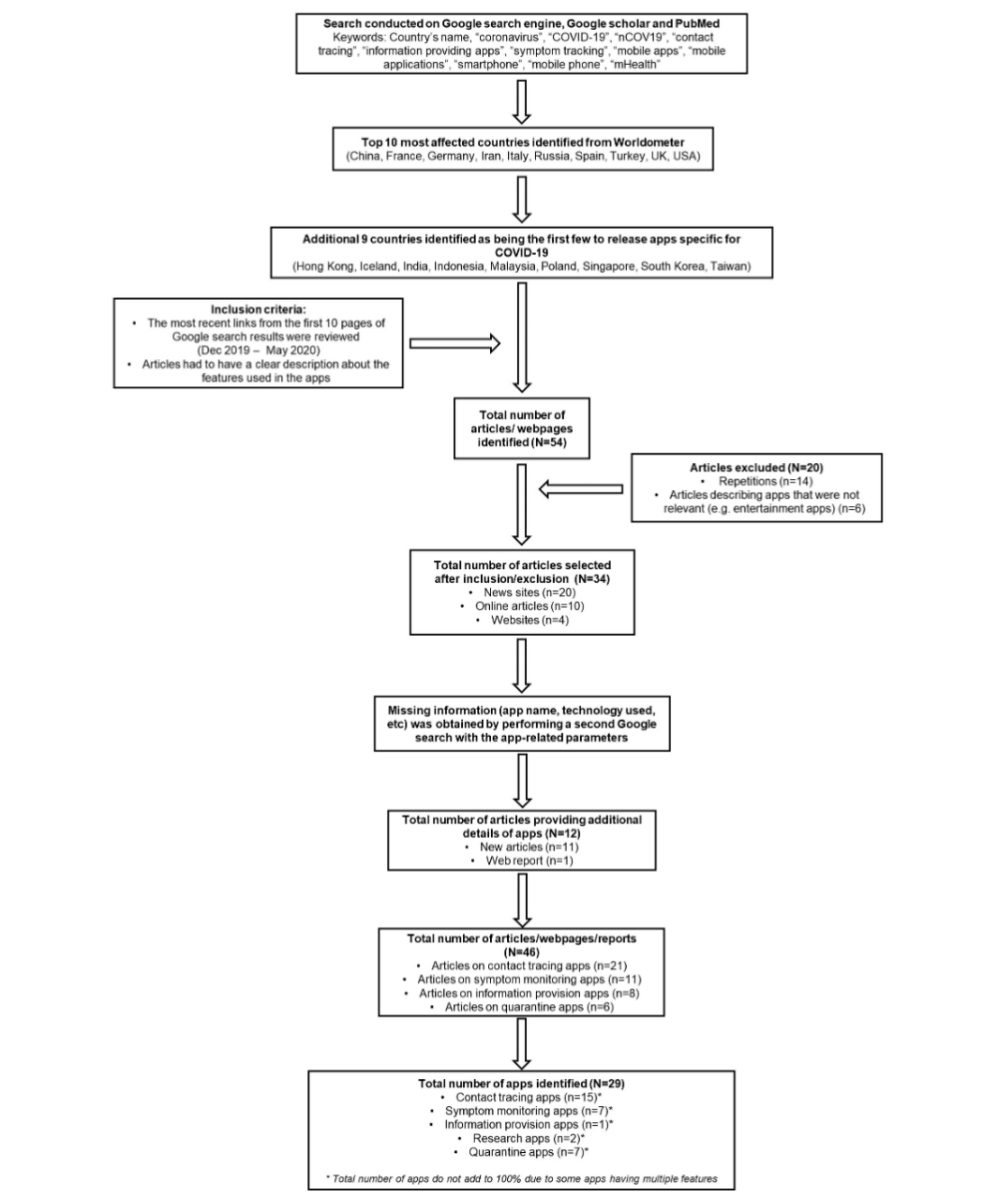
Flowchart of methodology. COVID-19: coronavirus disease; mHealth: mobile health.

A total of 46 articles that described apps from 19 different countries were reviewed (Figure 1). Most of the articles were from news sites, health care organizations, and government sites. The majority (13/19, 68.4%) of the countries studied either already had contact tracing apps or the apps were under development, followed by symptom monitoring apps (6/19, 31.6%). There was 1 app from Malaysia that had both symptom monitoring and information providing abilities (MySejahtera) [31]. Some countries like Italy, France, Germany, and 1 app from Malaysia were in the process of developing apps for contact tracing [31-34]. There was 1 global app found in the review [35].

Of all the apps evaluated (N=29), there were 14 (48%) apps on contact tracing alone, 1 (3%) app with both contact tracing and quarantine features, and 6 (21%) apps purely for enforcing quarantine (n=15 for contact tracing, n=7 for quarantine). Similarly, there were 5 (17%) apps on symptom monitoring alone, 1 (3%) app having both symptom monitoring and information provision features, 1 (3%) app with symptom monitoring feature and for research purposes, and 1 (3%) app was solely for research purposes (Table 1). There were 20 (69%) apps released from governments, 3 (10%) from private organizations, and 3 (10%) from universities. There were 3 (10%) apps that did not provide information about their source; 4 (14%) were web-based, 6 (21%) were available on only Android or iOS, and 10 (34%) were available on both platforms. There were 12 apps (41%) that did not provide information on their platform availability. In terms of the technology used, 10 (34%) apps used Bluetooth for collecting data, 12 (41%) apps used GPS, and 12 (41%) used other forms of data collection such as manual input of details and questionnaires.

**Table 1:** Mobile health apps related to COVID-19^a^ (N=29).

Country, App name		Organization/institution	Platforms	Purpose					Technology/method used to collect data		
				Contact tracing (n=15)	Symptom monitoring (n=7)	Information provision (n=1)	Research (n=2)	Quarantine (n=7)	Bluetooth (n=10)	GPS (n=12)	Others (n=12)
**China**											
	Beijing Cares [36,37]	Government_b_	N/A^c^	✓							Input of daily temperature
**France**											
	Under development [33]	Government	N/A	✓					✓		N/A
**Germany**											
	Corona-Datenspende [14,15]	Government	iOS^d^ and Android^e^		✓		✓				Smartwatch monitoring
	Under development [34]	Government	N/A	✓							N/A
**Hong Kong**											
	StayHomeSafe [29,38]	Government	iOS and Android					✓	✓	✓	Includes a wearable device and uses Wi-Fi and geospatial signals
**Iceland**											
	Rakning C-19 [39,40]	Government	iOS	✓						✓	N/A
**India**											
	Corontine [41,42]	University^f^ (Indian Institute of Technology)	Android					✓		✓	N/A
	GoCoronaGo [43]	University (Indian Institute of Science)	N/A	✓				✓	✓	✓	N/A
	Aarogya Setu [44]	Government	N/A	✓					✓		N/A
	COVID-Locator [45]	N/A	N/A	✓						✓	N/A
**Indonesia**											
	PeduliLindungi [46]	Government	N/A	✓					✓		N/A
**Iran**											
	AC19 [47]	Government	Android		✓					✓	Questionnaire
**Italy**											
	Under development [32]	Government	N/A	✓					✓		N/A
**Malaysia**											
	Under development (MySejahtera) [31]	Government	N/A		✓	✓					Self-input
	MyTrace [48]	Government	Android (iOS under development)	✓					✓		N/A
**Poland**											
	Home Quarantine [49,50]	Government	iOS and Android					✓		✓	Capturing a selfie
**Russia**											
	Social Monitoring [51]	Government	iOS and Android					✓		✓	N/A
**Singapore**											
	TraceTogether [7]	Government	iOS and Android	✓					✓		N/A
	COVID-19 Symptom Checker [13]	Government	Web^g^		✓						Questionnaire
**South Korea**											
	Corona 100m [30,52-54]	Private organization^h^	iOS and Android	✓						✓	Government surveillance data (ATM^i^ transactions and surveillance data)
	Self-quarantine Safety Protection [55,56]	Government	iOS and Android					✓		✓	N/A
**Spain (Community of Madrid)**											
	AsistenciaCOVID-19 [57,58]	Government	Web, iOS, and Android		✓						Questionnaire
**Taiwan**											
	Not available [59,60]	Government	N/A					✓			Mobile signals
**Turkey**											
	Corowarner [61]	Government	N/A	✓					✓	✓	N/A
**United Kingdom**											
	C-19 COVID Symptom Tracker [62-64]	Private organization	iOS and Android		✓						Self-reporting
	Under development [65]	N/A	N/A	✓							N/A
	COVID-19 Sounds [66,67]	N/A	Web and Android				✓				N/A
**United States**											
	Private Kit: Safe Paths [68,69]	University (Massachusetts Institute of Technology)	iOS and Android	✓					✓	✓	N/A
**Global**											
	COVID-19 Screening Tool [35]	Private organization (Apple)	Web and iOS		✓						Questionnaire

^a^COVID-19: coronavirus disease.

^b^n=20.

^c^N/A: not applicable.

^d^n=13.

^e^n=14.

^f^n=3.

^g^n=4.

^h^n=3.

^i^ATM: automated teller machine.

### Contact Tracing Apps

There were many varieties of contact tracing apps. China was the first country to develop an app specific for contact tracing by using sophisticated tracking and surveillance methods [36,37]. They involved tracking of infected individuals and their contacts, while others were allowed to carry on with their normal lives [38]. Other countries followed suit after a study conducted by the University of Oxford suggested that the release of contact tracing apps played a major role in decreasing the spread within the community [70]. Malaysia, Singapore, India, Indonesia, and Iceland were fast at developing individual apps for contact tracing [7,39-41,43-46,48]. On the other hand, Italy, Germany, France, and the United Kingdom had apps in the development stage during the period of this review [32-34,65].

Of the 15 contact tracing apps analyzed, 6 (40%) apps had used GPS technology. South Korea’s Corona 100m app used data such as the user’s last GPS history and a range of information from government information systems, surveillance footage, and credit card transactions [30,52-54]. The most common method for contact tracing apps was Bluetooth (n=9, 60%). These apps anonymously notified healthy individuals if they had been in close contact with an infected individual. This was in contrast to other techniques, such as the one used by China, using strict surveillance methods that raised privacy concerns since the technologies could track the person’s location and obtain personal data as well [36,37]. GPS and Bluetooth were used in combination by three contact tracing apps released by the United States, India, and Turkey [43,61,68,69]. All these apps were either supported by the government or from recognized health organizations after restrictions were imposed by the Apple and Google Play Stores.

### Quarantine Apps

In addition to contact tracing apps, various countries have also come up with quarantine apps to ensure that quarantine measures are being followed. For example, geofencing apps enforce the quarantine by using mobile phone signals and GPS to track the movements of users. The concept is to create a virtual fence around people’s houses so that when they disobey the regulations and go outside their houses, the authorities will be notified [43,71]. One of the countries that has adopted this technique is India [41,42,71]. Taiwan also uses the same technology to geofence affected individuals who are required to self-isolate or quarantine at home [59]. The geofencing app works by using base station triangulation, which is not as precise as GPS, but it provides the location with an accuracy of 300 meters. Quarantined individuals are assigned a social worker who calls and checks on them twice a day. If unresponsive, the police are notified and will then visit their house [60]. In addition, Taiwan has also accepted the help of its citizens to develop tools to solve issues like sourcing and distributing face masks to avoid shortage in the affected areas [62].

In Hong Kong, quarantine is enforced by providing a wearable device (wristband) and a mobile app to people who arrive from other countries. The wristband is scanned and integrated to the app upon installation, and it works by using GPS, Bluetooth, Wi-Fi, and geospatial signals in the neighborhood to determine position. If there is a difference in these signals, the app notifies its user and a quarantine officer [29,38]. When it was released, the public had doubts as to whether the wristband technology could work effectively, since it was paper-like and did not look robust. However, the majority adhered to wearing the wristbands because they believed that it would help them prevent the spread of the virus [72].

Other countries that have enforced quarantine apps include South Korea, Russia, and Poland. For example, the Self-Quarantine Safety Protection app by South Korea uses GPS to track quarantined individuals. Individuals who opt out of using the app are monitored by calls twice a day from an assigned quarantine officer [55,56]. On the other hand, Moscow has also made it mandatory for individuals who tested positive for the coronavirus to download and install the Social Monitoring quarantine app, which uses GPS to monitor movement [51]. Individuals who do not own smartphones will be provided with one that already has the app downloaded so that they can be tracked. In contrast, in addition to GPS tracking, Poland’s Home Quarantine app also requires the quarantined individual to take a selfie when randomly prompted to ensure that the individual is at their residence. Upon release of the app in Poland, people had the option to either use this app or agree to be visited by a police officer every day, but it was later made mandatory for everyone under quarantine [49,50]. According to a user of the app, she felt angry and stressed because she missed the alerts several times early in the morning and the police checked up on her at her home. In the second week, the number of alerts “doubled” and drained her phone battery. Furthermore, she became more stressed as she felt that she had to be on standby, even during a shower [73].

### Symptom Monitoring Apps

One of the global apps for symptom monitoring is the iPhone and web-based COVID-19 Screening Tool app developed by Apple [35]. This is available for all iPhone users, irrespective of their location. Furthermore, countries such as Spain, the United Kingdom, Germany, Singapore, and Malaysia have also developed symptom monitoring apps [13-15,31,57,66,67]. These apps identify if the user is experiencing symptoms related to COVID-19. If the user’s responses indicate that the user may have COVID-19, they are provided with simple management advice to follow. Generally, these apps pose a series of diagnostic questions that include symptoms like fever, type of cough, body aches, contact with any infected individual, and recent travel, among others, which help identify via a back end algorithm whether the user is suspected to have COVID-19. If the user is suspected to be infected, these apps will generally provide information on what to do in that scenario (eg, wearing a face mask to reduce the spread and providing information about nearby hospitals). For example, the United Kingdom’s symptom monitoring app, called C-19 COVID Symptom Tracker was made by a private developer [62-64]. It was highly welcomed by the public with 2,979,018 contributors as of May 6, 2020 [62]. The app was also helpful in identifying that 1 in 10 people in the United Kingdom had coronavirus symptoms [74]. In addition, Spain’s app could also alert officials on whether quarantine measures were being followed by the public in an area [58].

An advanced variation of symptom monitoring apps is Germany’s Corona-Datenspende app, which uses smartwatches or smart bands to collect data on the user’s biometrics such as temperature and pulse that are then assessed for possible COVID-19 infections [14]. Similarly, the COVID-19 Sounds App is a web-based app developed in the United Kingdom that is able to record a user’s cough sounds and detect whether the user is infected with the coronavirus based on machine learning of their cough sounds [66].

Among all the symptom monitoring apps reviewed in this study, besides the apps from Germany and the United Kingdom that were able to automatically monitor and record the patient’s health parameters, all of the other apps involved manual recording of symptoms or answering questionnaires provided by the app. Another app, called AC19, was released by the Iranian government in the Android app store for symptom monitoring but was later found to be using GPS technology to track suspected infected individuals. This app was found to be linked to a suspicious app developer company that had a history of developing other apps that collected and provided data to Iranian intelligence agencies. The app was later banned from the Google Play store but is still available through the developer’s website and other third-party app stores [47].

### Information Providing Apps

Various governmental organizations and health agencies have used social media platforms like Facebook, WhatsApp, Instagram, and Twitter to provide specific information about COVID-19 to the public. Although these platforms have app functionalities, Facebook, Instagram, and Twitter can also be used on a browser, and thus, information provision is not just limited to mobile app users. WhatsApp has been widely popular and is one of the main methods of providing information on COVID-19 in many countries. For example, government agencies from Australia, India, Singapore, and the United Kingdom harnessed the WhatsApp platform by developing their own chatbots to disseminate information to their citizens on the country’s COVID-19 situation and local measures taken during this pandemic [17-19,75,76]. However, in response to the infodemic that has surrounded COVID-19, it was necessary to regulate the information that was spread by users through WhatsApp [75]. WhatsApp also collaborated with the WHO and developed an information providing tool, which works by users messaging a designated number to request for information about COVID-19, such as infection numbers, hygiene practices, and locations of medical centers, among others [16,77]. The GovTech Agency in Singapore also developed its own artificial intelligence (AI) tool on WhatsApp to translate official news from English to other languages such as Chinese, Malay and Tamil to disseminate relevant information to its citizens [76]. It works similar to the WHO bot that provides a set of programmed responses that are updated with the information that has been requested by users. Malaysia has also proposed its own MySejahtera app, which is still under development and will include information provision features such as a hotline number and a Virtual Health Advisory along with symptom monitoring features [31].

## Discussion

### Principal Findings

From our review, contact tracing, symptom monitoring, and information providing apps were the key types of apps that had been developed for the management of COVID-19, with the majority being developed by health organizations and governments. To combat misinformation surrounding COVID-19, many organizations, including the two major mobile app players in the market—Apple and Google—made efforts to curate the COVID-19–related apps available in the app stores. Apps that provided misleading information were banned from their respective app stores, resulting in apps that were more credible and developed by established health care organizations and governments [25].

Not surprisingly, most of the apps identified from our review were for contact tracing. These apps were developed to alleviate the time and resources required for the manual contact tracing process, which could be channeled to other resources instead. However, a key issue for developers was to support information collection without compromising user privacy. The advantage of using GPS surveillance methods was the accuracy of the user’s identification. China’s method of data collection was deemed to be intrusive, as it invaded the privacy of individuals. However, this method was effective in identifying individuals who had breached the quarantine laws. Hence, it was welcomed by the general public in China, and they supported the government on its efforts to reduce the transmission of infection. Although this method could work in China, it could not be replicated in other countries due to the differences in their political and cultural stances on privacy [78]. Therefore, other countries explored and employed other data collection methods that were more mindful of privacy issues. Bluetooth-enabled contact tracing was the most popular method, since only when users had crossed paths would they have been detected, and data was not transferred and stored to any online server. A well-designed Bluetooth-enabled contact tracing app was first developed by Singapore, which was later shared to the rest of the world by making its development code open source [10]. This helped other countries roll out similar apps at a faster rate. Germany used an interesting and different approach of wearable devices for the automatic monitoring of vitals. The public acceptance of this technology is yet to be studied.

Although the responses to contact tracing apps were welcomed by most countries, it is still early to determine whether they are really effective in limiting the spread of the coronavirus [79]. It has been suggested that ~80% of the population needs to install such apps for it to be effective [80]. This is a concern because highly susceptible populations for COVID-19, such as older adults, are not adept with technology [81]. Furthermore, the majority of the contact tracing apps only register nearby users every 5 minutes and, hence, may have the possibility of missing out individuals. The range of Bluetooth is also farther than the recommended 1.5 meters advised by the WHO, thus giving rise to the possibility of over reporting the number of cases, especially in multistory buildings where the Bluetooth signal can pass through the walls [81]. Lastly, a Bluetooth-based contact tracing app will stop working altogether when the user opens a game app such as “Candy Crush.” Despite these problems, authorities are still advocating these apps even though the uptake of such apps are low, as it can play a role in benefiting the population to some extent [79,82].

Instead of having contact tracing apps specific to each country, a joint effort to develop a universal app for contact tracing may be more beneficial, since useful trends on the COVID-19 spread among the different countries can be identified and compared by international organizations such as the WHO. Hypothetically, the codes for developing such a universal app can be made open sourced and modified to suit each individual country’s needs and privacy laws. In fact, Apple and Google are collaborating to develop a universal app in the near future for contact tracing [83]. Their proposed method is to use Bluetooth to develop the app, similar to the way that Singapore had created their contact tracing app. However, the intended app will not use GPS data and will not store information online so that it can address the privacy concerns of many individuals. The Bluetooth app uses the “decentralized” approach since there is no recording of information into a back end database; thus, it is safer in terms of privacy because there is less risk of hackers accessing sensitive information. However, the disadvantage is that it may be difficult to enforce downloads and use of such apps [84]. On the other hand, the United Kingdom had opted for a “centralized” approach, where information would be stored and analyzed online, and notifications sent out based on interactions [84]. Although the centralized approach would pose a privacy risk, officials justified that this data would be helpful to identify trends of the disease spread [84]. However, there was concern regarding privacy by the general public, and the app did not function properly on iPhone devices during testing. Therefore, the United Kingdom government has subsequently decided to use the Apple and Google Bluetooth app to aid in its development, resulting in the app being decentralized as well [85].

Symptom monitoring apps were also useful in identifying disease trends and possible infection zones. Although most countries developed symptom monitoring apps based on manual input of symptoms and questionnaires, the symptom monitoring app developed in Germany could automatically collect the user’s vitals such as temperature and pulse, thus identifying patients who are symptomatic and possible infection zones through an interactive map [15]. This pandemic has also rapidly enhanced the uptake of telehealth systems in many countries. When combined with the use of wearable devices, it may be possible to conduct home screening and remote monitoring of COVID-19 symptoms through other integrated features such as mobile doctors or telehealth systems.

Our review managed to identify certain apps that had integrated information providing features with the other contact tracing and symptom monitoring features. Another popular method to disseminate COVID-19–related information was through social media platforms such as WhatsApp. Given the high rates of social media use, using social media channels to provide factual COVID-19 information would likely ensure the rapid and widespread access to relevant health information [86]. Combined with the fact that these apps are harnessed by various governments to provide reliable information about COVID-19, information such as advice to follow and precautions to take to prevent or avoid the spread of COVID-19 can be disseminated to the public in “chunks” as an educational resource, as well as to clarify their doubts. By combining with other features, a multipurpose app that provides all information and services about COVID-19 will potentially be attractive to users and the public.

### Future of Pandemic Management

**Figure 2:**
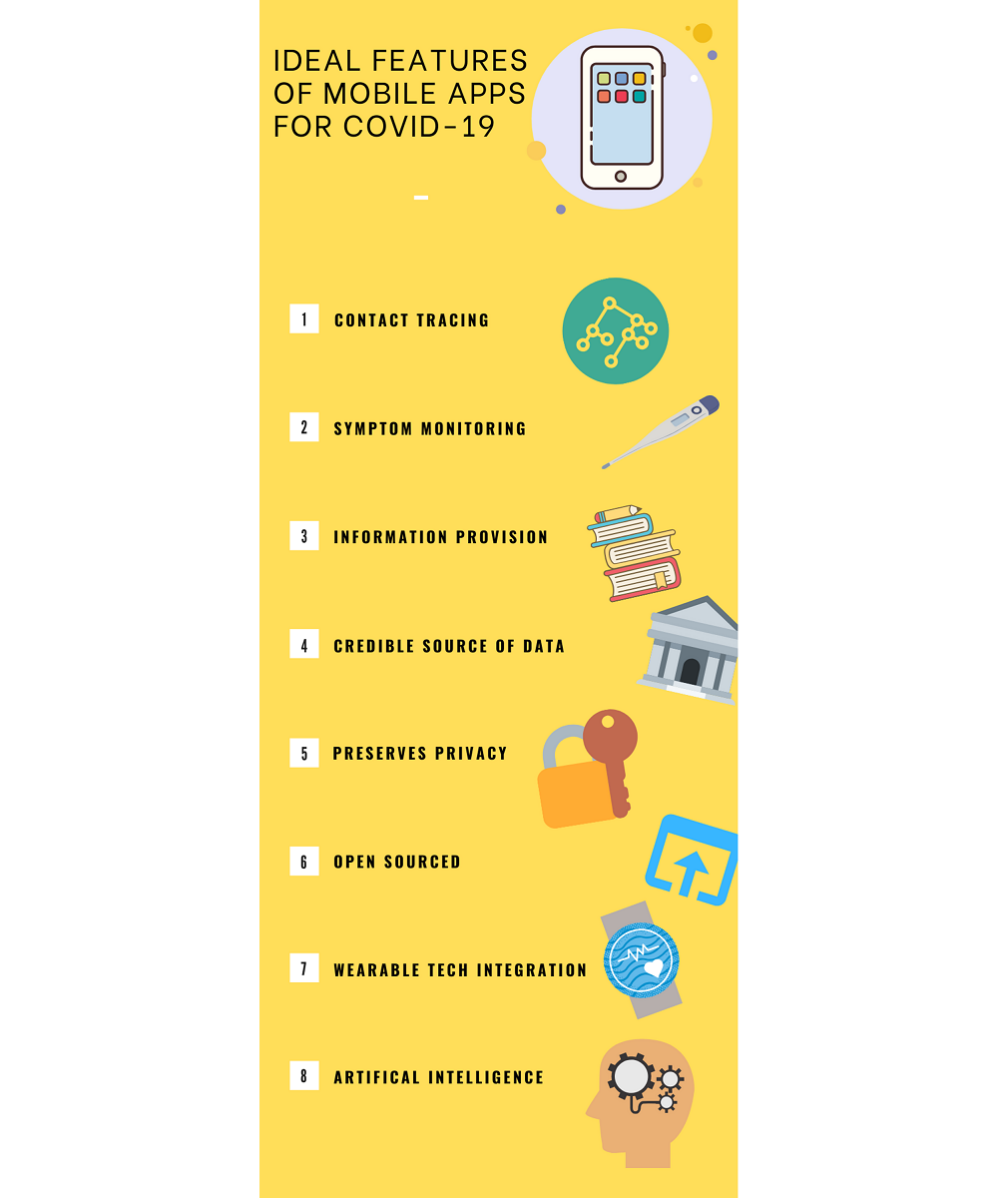
Ideal features of mobile apps for COVID-19. COVID-19: coronavirus disease.

To effectively tackle a pandemic such as COVID-19, a multipronged approach should be used. A proper contact tracing app that is implemented at the starting stages of the outbreak is important. Furthermore, authorities should make it a priority to advocate the uptake of contact tracing apps by the public by educating about the benefits of using such apps, perhaps through social media and public health campaigns. The Bluetooth-enabled contact tracing method is by far the most popular in terms of effectiveness and maintenance of privacy. The universal app that is currently being developed by Apple and Google can help limit the spread on a larger scale, as the reach of such an app will be greater and can provide an opportunity to prepare for similar future outbreaks internationally. Figure 2 suggests some features that should be available in mobile apps for COVID-19. Other important functionalities that can be integrated into these contact tracing apps include features for automatic symptom monitoring and information provision. The addition of these features will provide a more holistic public health approach in response to the situation. As technology advances, the symptom monitoring algorithm can be enhanced and tailored to the pandemic to improve its accuracy in diagnosis. Wearable devices such as smartwatches and smart bands will become more common and integrated with daily lives; thus, these can potentially aid in the vital monitoring of health statuses of vulnerable populations. Through machine learning and AI methods, automatic and rapid identification of suspected infections will become more accurate in the future. Lastly, consolidated information that is provided by credible organizations such as the WHO can avoid any unnecessary confusion as to which advice to follow in a pandemic situation. Governments can then adapt and tailor the information to suit their populations. With advancement in telehealth and mHealth systems, mobile doctors will be the way to go in situations like this where self-isolation is needed. However, with the advancements in technology, “digital humans” may potentially be the solution to reduce the burden of health care professionals in future pandemics [87].

### Limitations and Future Work

Since COVID-19 is a new outbreak and the situation is rapidly evolving, the articles in this review were mainly from news articles and online webpages. There were few peer-reviewed journal articles about mobile apps related to COVID-19. Furthermore, we could not conduct a thorough search of the Google and Apple apps stores as most of the apps were country-specific; thus, they could not be downloaded and evaluated. Hence, we could only obtain information from the news articles and webpages that were found, but the details on the various apps were also not comprehensive in these articles. In addition, the databases searched (Google Search Engine, Google Scholar, Scopus, and PubMed) might not have been able to provide information on certain apps due to country restrictions, for example, apps from China. Another limitation was that sources in languages other than English were also not included in this review. From these sources, we could only collate apps that were either already available to the general public or still under development during the period of review. We advocate that a more comprehensive review should be done in the near future when the situation is stabilized, possibly post–COVID-19. Future work on how effective these apps were in decreasing the coronavirus spread should also be undertaken. This will help identify apps and features that are beneficial in future pandemics.

### Conclusion

This review has identified a variety of apps that may be potentially useful to curb the spread of COVID-19. The majority of the apps were for the purposes of contact tracing and symptom monitoring. However, these apps, especially those for contact tracing, can only be effective if they are advocated by the government and taken up by the community. Contact tracing at an early stage, along with proper hygiene and social distancing practices, remain the ideal way to deal with COVID-19. Governments can also benefit by encouraging their citizens to participate in their efforts to combat the pandemic, as in the case of Taiwan. In addition, the sharing of good practices across different countries, such as the case of Singapore, can enable governments to learn from each other so that effective strategies to combat and manage this pandemic can be developed to control the spread of the coronavirus.

## Abbreviations

AI: artificial intelligence
COVID-19: coronavirus disease
mHealth: mobile health
PRISMA: Preferred Reporting Items for Systematic Reviews and Meta-Analyses
SARS-CoV-2: severe acute respiratory syndrome coronavirus 2
WHO: World Health Organization
